# Androgen Deprivation Therapy and the Risk of Newly Developed Dry Eye Syndrome in Patients with Prostate Cancer: A Nationwide Nested Case–Control Study in the Republic of Korea

**DOI:** 10.3390/jcm13175314

**Published:** 2024-09-08

**Authors:** Jinhyung Jeon, Jee Soo Ha, Hye Sun Lee, Soyoung Jeon, Ho Sik Hwang, Daeho Kim, June Seok Kim, Byeong Seon Kim, Min Kim, Kang Su Cho

**Affiliations:** 1 Department of Urology, Prostate Cancer Center, Gangnam Severance Hospital, Yonsei University College of Medicine, Seoul 03722, Republic of Korea; jun1644@yuhs.ac (J.J.); rlaeogh93@gmail.com (D.K.); idkjs12@yuhs.ac (J.S.K.); 2 Department of Urology, Seoul Hyocheon Foundation Medical Corporation H Plus YangJi Hospital, Seoul 08779, Republic of Korea; eng.zsu@gmail.com; 3 Biostatistics Collaboration Unit, Yonsei University College of Medicine, Seoul 03722, Republic of Korea; hslee1@yuhs.ac (H.S.L.); jsy0331@yuhs.ac (S.J.); 4 Department of Ophthalmology, Yeouido St. Mary’s Hospital, College of Medicine, The Catholic University of Korea, Seoul 06591, Republic of Korea; huanghs@daum.net; 5 Department of Urology, Severance Hospital, Yonsei University College of Medicine, Seoul 03722, Republic of Korea; urokim92@gmail.com; 6 Department of Ophthalmology, Institute of Vision Research, Gangnam Severance Hospital, Yonsei University College of Medicine, Seoul 03722, Republic of Korea; minkim76@yuhs.ac; 7 Center of Evidence Based Medicine, Institute of Convergence Science, Yonsei University, Seoul 03722, Republic of Korea

**Keywords:** androgens, androgen deprivation therapy, case–control studies, dry eye syndromes, prostate neoplasms

## Abstract

**Background:** We aimed to evaluate the association between androgen deprivation therapy (ADT) and newly developed dry eye syndrome (DES) in patients with prostate cancer. **Methods:** A nested case–control study was conducted. From the nationwide claims database of the Republic of Korea, 125,005 patients were included in the final analysis. Cases were defined as those newly diagnosed with DES during follow-up, and 12,654 patients were identified. The cases were matched with controls in a ratio of 1:4. Odds ratios (ORs) for newly developed DES associated with ADT were estimated using conditional logistic regression. **Results:** After matching, 7499 cases and 29,996 controls were selected. ADT was associated with a reduced risk of newly developed DES in patients with prostate cancer compared to no ADT (OR = 0.875; 95% confidence interval, 0.825–0.927; *p* < 0.0001). An accumulated dose of ADT < 1 year was associated with a reduced risk of incidental DES (OR = 0.811; 95% CI, 0.751–0.875; *p* < 0.0001), and a duration of 1–2 years was also associated with a reduced risk (OR = 0.890; 95% CI, 0.802–0.986; *p* = 0.026). No association was observed with an ADT duration of ≥2 years. **Conclusions:** The use of ADT, especially for shorter durations (<2 years), was associated with a reduced risk of newly developed DES in S. Korean patients with prostate cancer.

## 1. Introduction

Androgens, the male sex hormones, play a crucial role in developing and maintaining masculine characteristics in reproductive tissues and contribute to the anabolic status of somatic tissues [1]. The normal development and maintenance of the prostate depend on androgens acting through the androgen receptor, which is also essential for the development and progression of prostate cancer [2]. In 1941, Huggins and Hodges first reported the beneficial effects of castration and estrogen injections in patients with metastatic prostate cancer [3]. Androgen deprivation therapy (ADT) is the primary treatment for advanced prostate cancer and is used as an adjuvant for the local treatment of high-risk diseases [4]. Due to the psychological and physiological side effects of ADT, it has evolved from orchiectomy to estrogens and, finally, to gonadotropin-releasing hormone (GnRH) agonists [5]. Although ADT can improve the survival of patients with prostate cancer, it can also cause significant side effects and reduce the quality of life, including decreased bone health, metabolic consequences, increased diabetes risk, cardiovascular events, gynecomastia, reduced penile/testis size, fatigue, hot flashes, cognitive changes, and anemia [6].

In ophthalmology, an increased risk of cataract has been reported as a side effect of ADT for prostate cancer [7]. Conversely, ADT is associated with a decreased risk of primary open-angle and normal tension glaucoma in Korean patients with prostate cancer [8,9]. Although ADT is a known risk factor for cardiovascular events, it does not appear to increase the risk of retinal vascular occlusion in patients with prostate cancer [10]. Recent studies have reported conflicting results regarding the effects of ADT on dry eye syndrome (DES) in patients with prostate cancer. One study found that the androgen levels were associated with significant changes in meibomian gland function in patients with prostate cancer undergoing ADT, recommending monitoring the ocular surface of these patients [11]. However, a population-based cohort study in Taiwan found that ADT did not increase the incidence of DES in patients with prostate cancer in either the short or long term [12]. Therefore, more research is required to draw solid conclusions regarding the effect of ADT on DES.

DES is one of the most common diagnoses seen by ophthalmologists and refers to a group of disorders of the tear film that result from reduced tear production or tear film instability [13]. DES, which can cause constant irritation, foreign body sensation, and blurred vision, is considered a serious health problem that can interfere with the ability to work and perform daily functions [14]. Androgen deficiency has been associated with DES in several studies [15,16,17]. Thus, we evaluated the association between ADT and the newly developed DES in patients with prostate cancer using a nationwide claims database in the Republic of Korea.

## 2. Materials and Methods

### 2.1. Ethics

This study was conducted in accordance with all applicable laws and regulations, good clinical practice guidelines, and ethical principles outlined in the Declaration of Helsinki. The Institutional Review Board of Gangnam Severance Hospital approved the study protocol (approval number: 3-2021-0307).

### 2.2. Database

The National Health Insurance (NHI) System in the Republic of Korea is a universal health coverage system that encompasses over 95% of Korean residents. The Health Insurance Review and Assessment Service (HIRA) collect claims data submitted by healthcare providers for reimbursement. HIRA contains data on healthcare services for approximately 50 million beneficiaries annually [18].

### 2.3. Study Cohorts

**Figure 1:**
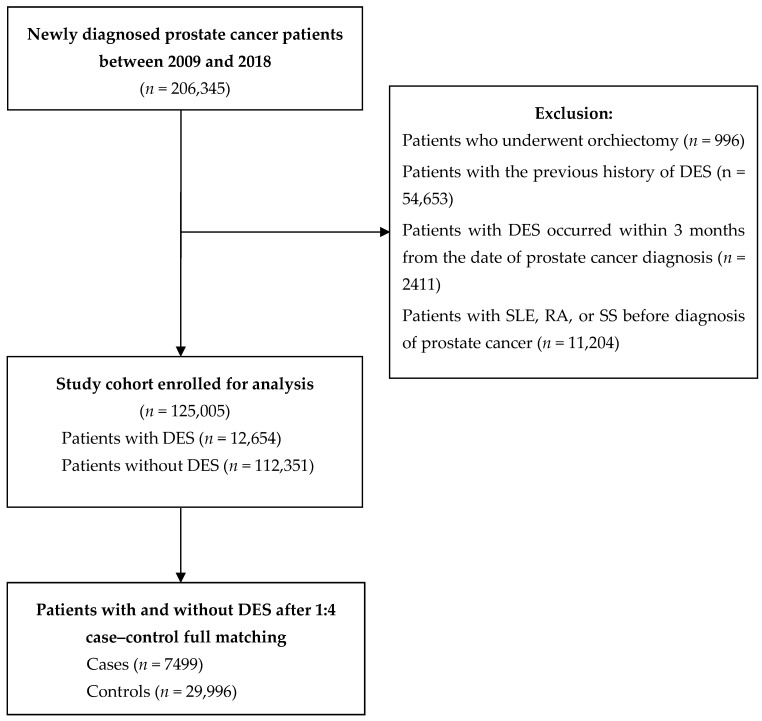
Flow diagram of the study cohort. RA, rheumatoid arthritis; SLE, systemic lupus erythematosus; SS, Sjogren’s syndrome.

Patients were identified using the International Classification of Diseases, 10th revision (ICD-10) codes. The study period extended from 1 August 2009 to 31 December 2018. In total, 206,345 patients newly diagnosed with prostate cancer (ICD-10 code: C61.0) were identified from the HIRA database. Exclusions were made for patients who underwent bilateral orchiectomy (*n* = 996), as the focus of our study was on the relationship between medical castration and DES, given that surgical castration is rarely employed in Korea. Additionally, patients with a history of DES (*n* = 54,653) or those experiencing DES within 3 months of a prostate cancer diagnosis were excluded (*n* = 2411). To enhance clarity regarding the relationship between ADT and DES, cases with a pre-diagnosis of inflammatory eye disease (*n* = 10,788); rheumatic disease involving the eye (including systemic lupus erythematosus, rheumatoid arthritis, or Sjögren’s syndrome) (*n* = 11,204); or vitamin A deficiency (*n* = 1288) were also excluded. ICD-10 codes used for identifying patients with a pre-diagnosis of inflammatory eye disease, rheumatic disease involving the eye, or vitamin A deficiency are detailed in Supplementary Table S1. The final cohort consisted of 125,005 patients (Figure 1).

### 2.4. Nested Case–Control Study

A nested case–control study was conducted to investigate the association between the cumulative duration of ADT and the development of new cases of DES. A case was defined as a patient who visited a medical institution, received a diagnosis of DES (ICD-10 code: H04.11), and was prescribed sodium hyaluronate lubricants (Anatomical Therapeutic Chemical [ATC] code: S01XA20). A total of 12,654 patients were identified during the follow-up period. Cases were fully matched in a 1:4 ratio with controls for all covariates that might be associated with the incidence of DES, identified using ICD-10 diagnostic codes at the date of prostate cancer diagnosis (Supplementary Table S2). The covariates included age, hypertension, diabetes, cardiovascular disease, hyperthyroidism, hypothyroidism, Parkinson’s disease, chronic kidney disease, cerebrovascular disease, dyslipidemia, and chronic pulmonary disease.

### 2.5. Definition of Therapy Exposure

ADT was defined as the administration of at least one dose of a GnRH agonist, antagonist, or anti-androgen therapy after the diagnosis of prostate cancer. The ADT duration was calculated as the sum of the action periods for each ADT preparation. In cases where the event occurred during ADT, the ADT duration was set as the sum of the action periods of the ADT preparation before the event occurred. The end of the follow-up period was defined as the date of the event or the date of the last valid inpatient or outpatient claim records.

### 2.6. Statistical Analysis

Statistical comparisons of continuous variables from patient baseline characteristics were performed using Student’s *t*-test, and categorical variables were compared using Pearson’s chi-square test. We utilized conditional logistic regression to estimate odds ratios (ORs) and 95% confidence intervals (CIs) for the association between DES and ADT. We explored whether there was a dose–response relationship between the duration of ADT and DES. Sensitivity analysis was conducted by repeating the same statistical procedure for the 1:1 and 1:10 case-to-control ratios. A *p*-value < 0.05 was considered statistically significant, and all statistical tests were two-sided. The SAS^®^ System for Windows^®^, version 9.4 (SAS Institute Inc., Cary, NC, USA), was used for all study analyses.

## 3. Results

After applying the exclusion criteria, 125,005 patients newly diagnosed with prostate cancer, with a median follow-up of 7.47 years, were eligible for the analysis (Figure 1). The baseline characteristics of the study cohort are presented in Table 1. Overall, 33,376 (26.8%) patients received ADT at some point during the follow-up, whereas 91,629 (73.3%) did not. A total of 12,654 patients were newly diagnosed with DES. The main analysis included 7499 cases and 29,996 controls. The baseline characteristics after case–control matching are presented in Table 2. The cases and controls were appropriately matched.

ADT was associated with a reduced risk of newly developed DES in patients with prostate cancer compared to non-ADT users (OR = 0.875; 95% CI, 0.825–0.927; *p* < 0.0001) (Table 3). An accumulated ADT duration of <1 year was linked to a reduced risk of newly developed DES (OR = 0.811; 95% CI, 0.751–0.875; *p* < 0.0001), while a duration of 1–2 years showed a similar association (OR = 0.890; 95% CI, 0.802–0.986; *p* = 0.026). However, no association was observed with ADT durations ≥2 years (OR = 1.023; 95% CI, 0.917–1.140; *p* = 0.687).

As a sensitivity analysis to assess the appropriateness of the 1:4 matching ratio, additional analyses were conducted at 1:1 and 1:10 ratios (Table 4 and Table 5). Both the 1:1 and 1:10 matching analyses revealed an association between ADT and a reduced incidence of newly developed DES. This association was most prominent when the duration of ADT was <1 year. However, in the 1:10 matching analysis, no significant association was observed between an ADT duration of 1–2 years and incidental DES. Using a 1:1 matching ratio preserved the number of cases (cases = 10,949; controls = 10,949), while employing a 1:10 matching ratio increased the overall number of analyses (cases = 3461; controls = 34,610). The results of the sensitivity analysis demonstrated a trend consistent with the main analysis, suggesting that a 1:4 full matching ratio maintained both the case count and statistical power.

## 4. Discussion

Patients with prostate cancer undergoing ADT may serve as an appropriate study population for investigating the association between androgen deficiency and DES. The present study found that ADT was associated with a lower risk of newly developed DES in patients with prostate cancer, particularly when the duration of ADT was <2 years. This finding contrasts with the results of a previous population-based cohort study in Taiwan, which indicated that ADT did not change the incidence of subsequent DES in either the short or long term [12]. Their study did not show statistically significant differences between the ADT and non-ADT groups in patients with prostate cancer; the adjusted HR (95% CI) were 0.980 (0.771–1.246) and 1.064 (0.855–1.325), respectively. Androgens are known to be essential for both normal prostate tissue and prostate cancer tissue, exerting a strong influence on the lipid composition in meibomian gland secretions through the androgen receptor. The androgen receptor is expressed throughout the eye, including the lacrimal gland, meibomian gland, cornea, and bulbar and fornical conjunctivae [2,19]. While androgen deficiency is generally regarded as a risk factor for DES, our study showed that ADT had a protective effect against incidental DES, particularly in the short term [20]. This suggests that the effects of ADT-induced androgen deficiency on eye health may differ from those of general androgen deficiency.

ADT inhibits the hypothalamic–pituitary–gonadal axis, leading to reduced testosterone production from the testes [21]. However, even at castration levels, androgen production from other sources such as the adrenal glands continues, resulting in testosterone levels similar to those found in women [21,22]. Consequently, it may be unlikely that the androgen levels achieved through ADT are sufficient to independently cause DES. Although androgen are typically known for their anti-inflammatory effects, they can exhibit proinflammatory properties in certain conditions, such as in polycystic ovary syndrome and acne [23,24]. The relatively short-term protective effect of ADT against incidental DES observed in our study, although not directly evidenced, may be related to adaptive hormonal changes that could potentially mitigate any proinflammatory effects and contribute to an ocular environment that protects against the development of DES. This protective effect might be transient, diminishing as the body fully adapts to the hormonal changes induced by ADT.

In addition to hormonal mechanisms, recent research suggests that microbiome changes might also play a role in modulating DES risk in patients undergoing ADT. An observational study indicates that ADT results in significant changes in the gut microbiome [25]. The ocular microbiome also varies, depending on different ocular diseases such as meibomian gland dysfunction and lacrimal dysfunction [26]. Additionally, conditions like Sjogren’s syndrome, diabetic retinopathy, glaucoma, macular degeneration, and infectious keratitis have been linked to gut microbiome alterations [27]. Although direct evidence is lacking, the systemic influence of the microbiome suggests a plausible pathway through which ADT could indirectly impact ocular health. These microbiome-related changes could reduce inflammation and create a temporary protective environment for the ocular surface.

DES is a multifactorial and self-perpetuating inflammatory disease with intrinsic factors such as aging, autoimmunity, and drying medications and extrinsic factors including a desiccating environment and exposure to irritants that can contribute to the inflammatory cycle [28]. Although DES can be classified into two types: dry eye with reduced tear production (aqueous-deficient) and dry eye with increased evaporation of the tear film (hyper-evaporative), the mixed form is more common [20]. This makes it difficult to ascribe a single cause to most cases of DES and highlights the importance of addressing all modifiable risk factors. According to the Tear Film and Ocular Surface Society International Dry Eye Workshop, androgen insufficiency is a well-known risk factor for DES and is associated with both aqueous-deficient and evaporative types [29]. That guideline reviewed several animal studies documenting the effects of orchiectomy or androgen treatment on the lacrimal glands, implicating androgen deficiency as a risk factor for aqueous-deficient DES. Although androgen insufficiency was associated with aqueous-deficient-type DES, it could indirectly promote the progression of Sjögren’s syndrome, and most studies available in this literature seemed to be more concentrated on meibomian gland dysfunction resulting in androgen deficiency [30]. Patients with complete androgen insensitivity syndrome, who have dysfunctional androgen receptors, exhibit significantly altered lipid fractions in meibomian gland secretions [17]. However, no linear association was found between the DES and androgen levels in a study involving 263 older male patients [15]. They only revealed a weak correlation between higher androstenedione levels and healthier global, lipid, and aqueous tear film parameters. The lack of a clear association between androgen levels and DES suggests that there may be no single, specific cutoff value for androgen levels associated with DES.

Previous studies found that DES is more common in women than in men, suggesting that sex-related differences are largely attributable to the effects of sex steroids, hormones, sex chromosome complements, sex-specific autosomal factors, and epigenetics [31]. The higher prevalence of DES signs and/or symptoms in women has been associated with various systemic conditions, including Sjögren’s syndrome; complete androgen insensitivity syndrome; premature ovarian failure; polycystic ovary syndrome; and physiological changes related to pregnancy, lactation, menstruation, and menopause [32]. After menopause, the production of estrogen and testosterone by the ovaries is markedly reduced. There is substantial evidence indicating that androgens and estrogens are implicated in DES [33]. Female patients with DES tend to be more symptomatic than male patients, especially when only mild or moderate signs are present [34]. However, the role of estrogen in DES remains unresolved and may be exerted indirectly by antagonizing the action of androgen, impacting meibomian gland physiology and stimulating function, and suppressing keratinization [11,32].

In a randomized placebo-controlled study, estrogen supplementation worsened the ocular symptoms in postmenopausal women with DES, while testosterone therapy showed no apparent impact on the symptoms [33]. However, a subgroup analysis revealed increased tear secretion in the testosterone plus estradiol combination group (*p* = 0.03) and a strong association between elevated serum androgen levels and improved tear stability in the testosterone group (*p* = 0.01). In contrast, a randomized, controlled, double-blind study in DES patients with androgen deficiency demonstrated that transdermal androgen was effective in relieving the symptoms and signs of DES, as well as improving the quality of life in aging patients, without serious side effects during short-term treatment [35]. These findings suggest that topical androgen therapy is a potential treatment option for patients with DES. It is important to note, however, that the sample sizes in both studies were relatively small (40 and 50 patients, respectively). Moreover, the latter study included only 12 male patients, making it challenging to generalize the results.

The strength of this study lay in the inclusion of a large number of patients in the analysis through a nationwide cohort. Furthermore, all covariates potentially influencing the development of DES were meticulously matched to augment the reliability of the analysis. However, the study had some limitations. Firstly, reliance on HIRA data for identifying patients with DES using ICD-10 and ATC codes may have led to an underestimation of DES cases. Nevertheless, over-the-counter medications are typically considered for relatively mild symptoms, while individuals with pronounced symptoms are likely to seek a diagnosis and receive prescriptions for lubricants at hospitals due to the accessibility of healthcare in South Korea. This approach better reflects the actual impact of ADT on newly developed DES. Secondly, our findings were derived from a population in South Korea, which may limit the generalizability of the results to other populations. Differences in healthcare practices, genetic predispositions, and environmental factors across countries could influence the applicability of our findings elsewhere. Future studies should consider these variables and potentially include diverse populations to better understand the global implications of ADT on DES. Finally, the study failed to explain the paradoxical protective effect observed among patients with prostate cancer undergoing ADT, despite androgen deficiency being a recognized risk factor for DES. Additional research is required to elucidate the underlying mechanisms of the protective effect of ADT on DES and to understand why this effect is only evident with short-term ADT.

## 5. Conclusions

ADT was not found to be associated with an increased risk of newly developed DES in Korean patients with prostate cancer. Interestingly, ADT exhibited a paradoxical protective effect against DES in patients with prostate cancer, particularly when the duration of ADT was <2 years. Further research is warranted to unravel the mechanisms that counteract the exacerbating effects of ADT-induced androgen deficiency in patients with prostate cancer in DES.

## Figures and Tables

**Table 1:** Demographic characteristics of the entire cohort.

Variables	Overall (*n* = 125,005)	Case (*n* = 12,654)	Control (*n* = 112,351)	*p*-Value
ADT use				<0.0001
never	91,629 (73.30)	10,106 (79.86)	81,523 (72.56)	
ever	33,376 (26.70)	2548 (20.14)	30,828 (27.44)	
Cumulative duration of ADT				<0.0001
none	91,629 (73.30)	10,106 (79.86)	81,523 (72.56)	
<1 year	13,092 (10.47)	1180 (9.33)	11,912 (10.60)	
1~2 years	7681 (6.14)	656 (5.18)	7025 (6.25)	
≥2 years	12,603 (10.08)	712 (5.63)	11,891 (10.58)	
Hypertension	43,982 (35.18)	3676 (29.05)	40,306 (35.88)	<0.0001
Diabetes	38,655 (30.92)	3423 (27.05)	35,232 (31.36)	<0.0001
Cardiovascular disease	26,660 (21.33)	2528 (19.98)	24,132 (21.48)	<0.0001
Hyperthyroidism	2541 (2.03)	233 (1.84)	2308 (2.05)	0.1075
Hypothyroidism	3423 (2.74)	333 (2.63)	3090 (2.75)	0.4378
Parkinson’s disease	1237 (0.99)	85 (0.67)	1152 (1.03)	<0.0001
Chronic kidney disease	4797 (3.84)	419 (3.31)	4378 (3.90)	<0.0001
Cerebrovascular disease	14,193 (11.35)	1135 (8.97)	13,058 (11.62)	<0.0001
Dyslipidemia	48,331 (38.66)	4798 (37.92)	43,533 (38.75)	0.069
Chronic pulmonary disease	44,593 (35.67)	4253 (33.61)	40,340 (35.91)	<0.0001
Age, years	66.423 ± 10.569	64.240 ± 9.744	66.669 ± 10.630	<0.0001

ADT, androgen deprivation therapy.

**Table 2:** Demographic characteristics after 1:4 full matching.

Variables	Overall (*n* = 37,495)	Case (*n* = 7499)	Control (*n* = 29,996)	*p*-Value
ADT use				<0.0001
never	29,128 (77.69)	5958 (79.45)	23,170 (77.24)	
ever	8367 (22.31)	1541 (20.55)	6826 (22.76)	
Cumulative duration of ADT				<0.0001
none	29,128 (77.69)	5958 (79.45)	23,170 (77.24)	
<1 year	4376 (11.67)	770 (10.27)	3606 (12.02)	
1~2 years	2125 (5.67)	381 (5.08)	1744 (5.81)	
≥2 years	1866 (4.98)	390 (5.20)	1476 (4.92)	
Hypertension	10,205 (27.22)	2041 (27.22)	8164 (27.22)	>0.9999
Diabetes	9080 (24.22)	1816 (24.22)	7264 (24.22)	>0.9999
Cardiovascular disease	5395 (14.39)	1079 (14.39)	4316 (14.39)	>0.9999
Hyperthyroidism	0 (0.00)	0 (0.00)	0 (0.00)	>0.9999
Hypothyroidism	10(0.03)	2(0.03)	8(0.03)	>0.9999
Parkinson’s disease	5 (0.01)	1 (0.01)	4 (0.01)	>0.9999
Chronic kidney disease	105 (0.28)	21 (0.28)	84 (0.28)	>0.9999
Cerebrovascular disease	1375 (3.67)	275 (3.67)	1100 (3.67)	>0.9999
Dyslipidemia	12,600 (33.60)	2520 (33.60)	10,080 (33.60)	>0.9999
Chronic pulmonary disease	11,715 (31.24)	2343 (31.24)	9372 (31.24)	>0.9999
Age, years	64.609 ± 9.293	64.600 ± 9.303	64.612 ± 9.291	0.9192

ADT, androgen deprivation therapy.

**Table 3:** Relationship between ADT and newly developed DES in 1:4 full matching ^a^.

Variables	Cases (*n* = 7499) *n* (%)	Control (*n* = 29,996) *n* (%)	OR (95% CI)	*p*-Value
ADT use				
never	5958 (79.45)	23,170 (77.24)	Reference	
ever	1541 (20.55)	6826 (22.76)	0.875 (0.825–0.927)	<0.0001
Cumulative duration of ADT				
none	7509 (79.11)	29,256 (77.05)	Reference	
<1 year	978 (10.30)	4613 (12.15)	0.811 (0.751–0.875)	<0.0001
1~2 years	516 (5.44)	2236 (5.89)	0.890 (0.802–0.986)	0.026
≥2 years	489 (5.15)	1863 (4.91)	1.023 (0.917–1.140)	0.687

ADT, androgen deprivation therapy; DES, dry eye syndrome; OR, odds ratio; CI, confidence interval. ^a^ covariate: age, hypertension, diabetes, cardiovascular disease, hyperthyroidism, hypothyroidism, Parkinson’s disease, chronic kidney disease, cerebrovascular disease, dyslipidemia, and chronic pulmonary disease.

**Table 4:** Relationship between ADT and newly developed DES in 1:1 full matching ^a^.

Variables	Cases (*n* = 10,949) *n* (%)	Control (*n* = 10,949) *n* (%)	OR (95% CI)	*p*-Value
ADT use				
never	8743 (79.85)	8441 (77.09)	Reference	
ever	2206 (20.15)	2508 (22.91)	0.834 (0.779–0.893)	<0.0001
Cumulative duration of ADT				
none	8743 (79.85)	8441 (77.09)	Reference	
<1 year	1039 (9.49)	1290 (11.78)	0.758 (0.692–0.831)	<0.0001
1~2 years	558 (5.10)	643 (5.87)	0.827 (0.732–0.934)	0.0022
≥2 years	609 (5.56)	575 (5.25)	1.023 (0.903–1.159)	0.7175

ADT, androgen deprivation therapy; DES, dry eye syndrome; OR, odds ratio; CI, confidence interval. ^a^ covariate: age, hypertension, diabetes, cardiovascular disease, hyperthyroidism, hypothyroidism, Parkinson’s disease, chronic kidney disease, cerebrovascular disease, dyslipidemia, and chronic pulmonary disease.

**Table 5:** Relationship between ADT and newly developed DES in 1:10 full matching ^a^.

Variables	Cases (*n* = 3461) *n* (%)	Control (*n* = 34,610) *n* (%)	OR (95% CI)	*p*-Value
ADT use				
never	2692 (77.78)	26,486 (76.53)	Reference	
ever	769 (22.22)	8124 (23.47)	0.924 (0.846–1.009)	0.0801
Cumulative duration of ADT				
none	2692 (77.78)	26,486 (76.53)	Reference	
<1 year	423 (12.22)	4635 (13.39)	0.889 (0.795–0.994)	0.0384
1~2 years	184 (5.32)	2050 (5.92)	0.876 (0.745–1.030)	0.1095
≥2 years	162 (4.68)	1439 (4.16)	1.121 (0.937–1.340)	0.212

ADT, androgen deprivation therapy; DES, dry eye syndrome; OR, odds ratio; CI, confidence interval. ^a^ covariate: age, hypertension, diabetes, cardiovascular disease, hyperthyroidism, hypothyroidism, Parkinson’s disease, chronic kidney disease, cerebrovascular disease, dyslipidemia, and chronic pulmonary disease.

## Data Availability

The data used in this study were obtained from the National Health Insurance Service (NHIS) database, which contains all claims data from the Korean single-payer system. Access to the NHIS database is strictly limited to researchers for the purpose of public health research and is not publicly available due to ethical restrictions and personal information protection laws in South Korea. However, the data are available from the NHIS database (https://nhiss.nhis.or.kr) for researchers who meet the criteria for access to confidential data, accessed on 31 March 2022.

## Supplementary Materials

The following supporting information can be downloaded at: https://www.mdpi.com/article/10.3390/jcm13175314/s1, Supplementary Table S1: ICD-10 diagnostic codes used for exclusion. Supplementary Table S2: ICD-10 diagnostic codes of covariates.
