# Supplementary material for: Androgen Deprivation Therapy and the Risk of Newly Developed Dry Eye Syndrome in Patients with Prostate Cancer: A Nationwide Nested Case–Control Study in the Republic of Korea
Source: J Clin Med. 2024 Sep 8;13(17):5314. doi: 10.3390/jcm13175314 (PMC11395731; doi:10.3390/jcm13175314)
Supplement: Supplementary file 1 [file jcm-13-05314-s001.zip › jcm-3125715-supplementary.pdf]

Supplementary Table S1. ICD-10 diagnostic codes used for exclusion

Disease	ICD-10 code
Inflammatory disease of lacrimal gland	H04.0 Other and unspecified dacryoadenitis: H04.00, H04.01, H04.08<br>H04.1 Other disorders of lacrimal gland: H04.18<br>H04.3 Acute or other and unspecified inflammation of lacrimal passages: H04.30, H04.31, H04.32, H04.38<br>H04.4 Other and unspecified chronic inflammation of lacrimal passages: H04.40, H04.41, H04.42, H04.48<br>H04.8 Other disorders of lacrimal system<br>H04.9 Disorder of lacrimal system, unspecified
Systemic Lupus erythematosus	M32 Systemic lupus erythematosus, unspecified: M32.0, M32.1, M32.8, M32.9
Rheumatoid arthritis	M05.0, M05.3, M05.8, M05.9<br>M06.0, M06.1, M06.2, M06.3, M06.4, M06.8, M06.9
Sjogren's syndrome	M35.0 Sicca syndrome[Sjogren]<br>H19.3 Keratitis and keratoconjunctivitis in other diseases classified elsewhere<br>M35.8 Other specified systemic involvement of connective tissue<br>M35.9 Systemic involvement of connective tissue, unspecified
Vitamin A deficiency	E50.0, E50.1, E50.2, E50.3, E50.4, E50.5, E50.6, E50.7, E50.8, E50.9

Supplementary Table S2. ICD-10 diagnostic codes of covariates.

Disease	ICD-10 code
Hypertension	I10 (Primary hypertension): I10.1, I10.9<br>I15 (Secondary hypertension): I15.0, I15.1, I15.2, I15.8, I15.9
Diabetes	E10 (Type 1 diabetes mellitus): E10.0, E10.1, E10.2, E10.3, E10.4, E10.5, E10.6, E10.7, E10.8, E10.9<br>E11 (Type 2 diabetes mellitus): E11.0, E11.1, E11.2, E11.3, E11.4, E11.5, E11.6, E11.7, E11.8, E11.9<br>E12 (Malnutrition-related diabetes mellitus): E12.0, E12.1, E12.2, E12.3, E12.4, E12.5, E12.6, E12.7, E12.8, E12.9<br>E13 (Other specified diabetes mellitus): E13.0, E13.1, E13.2, E13.3, E13.4, E13.5, E13.6, E13.7, E13.8, E13.9<br>E14 (Unspecified diabetes mellitus): E14.0, E14.1, E14.2, E14.3, E14.4, E14.5, E14.6, E14.7, E14.8, E14.9
Cardiovascular disease	I20 (Angina pectoris): I20.0, I20.1, I20.8, I20.9<br>I21 (Acute myocardial infarction): I21.0, I21.1, I21.2, I21.3, I21.4, I21.9<br>I22 (Subsequent myocardial infarction): I22.0, I22.1, I22.8, I22.9<br>I24 (Other acute ischemic heart disease): I24.0, I24.1, I24.8, I24.9<br>I25 (Chronic ischemic heart disease): I25.0, I25.1, I25.2, I25.3, I25.4, I25.5, I25.6, I25.8 I25.9<br>I50 (Heart failure): I50.0, I50.1, I50.9
Hyperthyroidism	E05 (Thyrotoxicosis[hyperthyroidism): E05.0, E05.1, E05.2, E05.3, E05.4, E05.5, E05.8, E05.9
Hypothyroidism	E00 (Congenital iodine-deficiency syndrome): E00.0, E00.1, E00.2, E00.9<br>E03 (Other hypothyroidism): E03.0, E03.1, E03.2, E03.3, E03.4, E03.5, E03.8, E03.9
Parkinson's disease	G20 (Parkinson's disease)
Chronic kidney disease	N18 (Chronic kidney disease): N18.1, N18.2, N18.3, N18.4, N18.5, N18.9<br>N03 (Chronic nephritic syndrome): N03.0, N03.1, N03.2, N03.3, N03.4, N03.5, N03.6, N03.7, N03.8, N03.9<br>N05 (Unspecified nephritic syndrome): N05.0, N05.1, N05.2, N05.3, N05.4, N05.5, N05.6, N05.7, N05.8, N05.9

Cerebrovascular diseases	I60 (Subarachnoid hemorrhage): I60.0, I60.1, I60.2, I60.3, I60.4, I60.5, I60.6, I60.7, I60.8, I60.9<br>I61 (Intracerebral hemorrhage): I61.0, I61.1, I61.2, I61.3, I61.4, I61.5, I61.6, I61.7, I61.8, I61.9<br>I62 (Other nontraumatic intracranial hemorrhage): I62.0, I62.1, I62.9<br>I63 (Cerebral infarction): I63.0, I63.1, I63.2, I63.3, I63.4, I63.5, I63.6, I63.7, I63.8, I63.9<br>I64 (Stroke, not specified as hemorrhage or infarction)
Dyslipidemia	E78 (Disorders of lipoprotein metabolism and other lipidemia): E78.0, E78.1, E78.2, E78.4, E78.5
Chronic pulmonary disease	J41 (Simple and mucopurulent chronic bronchitis): J41.0, J41.1, J41.8<br>J43 (Emphysema): J43.0, J43.1, J43.2, J43.8, J43.9<br>J44 (Other chronic obstructive pulmonary disease): J44.0, J44.1, J44.8, J44.9<br>J45 (Asthma): J45.0, J45.1, J45.8, J45.9<br>J47 (Bronchiectasis)
